# Four-Wave Mixing Crosstalk Suppression Based on the Pairing Combinations of Differently Linear-Polarized Optical Signals

**DOI:** 10.1155/2014/243795

**Published:** 2014-05-04

**Authors:** Haider Abd, Norashidah Md. Din, M. H. Al-Mansoori, F. Abdullah, H. A. Fadhil

**Affiliations:** ^1^Centre for Communication Services Convergence Technologies, College of Engineering, Universiti Tenaga Nasional, Jalan Ikram-Uniten, 43000 Kajang, Malaysia; ^2^Faculty of Engineering, Sohar University, P.O. Box 44, PCI 311 Sohar, Oman; ^3^School of Computer Engineering, University Malaysia Perlis, Malaysia, 02600 Arau, Perlis, Malaysia

## Abstract

A new approach to suppressing the four-wave mixing (FWM) crosstalk by using the pairing combinations of differently linear-polarized optical signals was investigated. The simulation was conducted using a four-channel system, and the total data rate was 40 Gb/s. A comparative study on the suppression of FWM for existing and suggested techniques was conducted by varying the input power from 2 dBm to 14 dBm. The robustness of the proposed technique was examined with two types of optical fiber, namely, single-mode fiber (SMF) and dispersion-shifted fiber (DSF). The FWM power drastically reduced to less than −68 and −25 dBm at an input power of 14 dBm, when the polarization technique was conducted for SMF and DSF, respectively. With the conventional method, the FWM powers were, respectively, −56 and −20 dBm. The system performance greatly improved with the proposed polarization approach, where the bit error rates (BERs) at the first channel were 2.57 × 10^−40^ and 3.47 × 10^−29^ at received powers of −4.90 and −13.84 dBm for SMF and DSF, respectively.

## 1. Introduction

Four-wave mixing (FWM) is one of the phenomena that may lower the effectiveness of the transmitted signal in wavelength division multiplexing (WDM) systems under dense channel spacing and low chromatic dispersion. In a WDM system with equally spaced channels, the new frequencies generated by FWM will drop at the channel frequencies and will introduce crosstalk [1–3]. The FWM effect is a result of the change in the intensity dependence of the refractive index of optical fiber.

Few reports and methods have been proposed for solving the problems associated with FWM. The examples of such methods are the use of nonzero dispersion fibers, relatively low channel counts, and unequal channel spacing techniques [4–6]. However, dispersion causes the distortion of the transmitted signals and needs to be compensated to achieve a long-haul system. As the channel count increases, more channels have to be confined to the erbium-doped fiber amplifier gain band by reducing the channel spacing. This condition increases the FWM effects and has a negative effect on the FWM suppression methods. Increased channel separation would prevent the implementation of a dense WDM. Similarly, reducing the levels of FWM crosstalk by choosing unequal channel frequency spacing may not be a practical option because this technique also needs additional optical bandwidth.

By contrast, orthogonal polarization has recently been found to reduce the FWM crosstalk. The FWM time average power strongly depends on the relative polarization states of the mixing channels. The researcher has reduced the FWM by randomly adjusting the polarization state of the adjacent channels to be orthogonal to one another [7–10]. Nevertheless, adjusting the polarization state randomly will not surely reduce the FWM crosstalk in all optical channels. Furthermore, the bit error rate (BER) may be not improved in all users because the orthogonal polarization does not include all channel interactions.

In this work, we combined pairs of channels with different polarizations. The first channel was polarized by a linear polarization of 45°, while the second channel was polarized at 90° away by a linear polarization of (45° + 90°). Both of the polarized channels were combined using a polarizer combiner. The proposed technique was investigated in both single-mode fiber (SMF) and dispersion-shifted fiber (DSF) with a 70 km fiber length and four channels. Through this approach, the FWM crosstalk significantly reduced and a good improvement was observed in system performance. The results confirm the robustness of the polarization technique against the FWM crosstalk and show that the FWM crosstalk has no dangerous influence on the system performance, even at a high value of input power.

## 2. System Description and Theoretical Background

**Figure 1:**
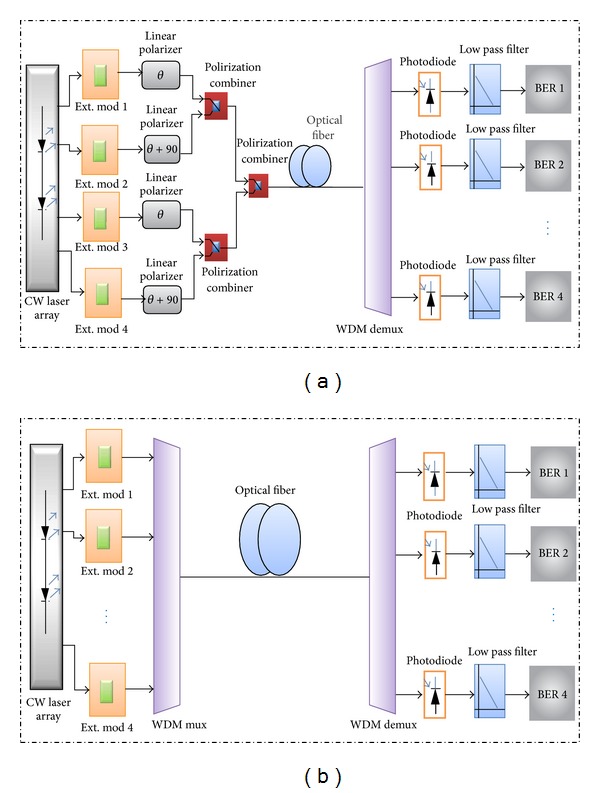
Optical system simulation configuration (a) proposed system and (b) conventional system.

Figures 1(a)-1(b) describe the proposed and conventional system configuration of the transmitter and receiver. At the transmitter part, the array of continuous wave lasers (L_1_–L_4_) is used to generate the carrier signal. The frequency of the first user is set to 193 THz, and the spacing between each user is 100 GHz. Each user is modulated with a 10 Gb/s data rate. Therefore, the total data rate of the system was 40 Gb/s. The array laser sources are connected to an external modulator. The external modulator comprised a Pseudo-Random Bit Sequence (PRBS), which is connected to a pulse generator to modulate the optical signals using an NRZ modulation format. It is then connected to the Mach-Zehnder modulator (MZM), which acts as an intensity modulator.

In the proposed system simulation, each two channels are linearly polarized 90° apart and then combined together. As shown in Figure 1(a), the first channel is polarized using a linear polarization of (*θ*), while the second channel is polarized using a linear polarization of (*θ* + 90°). Each of the two channels is combined using a polarizer combiner that combines the two input signals to one output port. The polarization angle has been selected at *θ* = 45°. Then, the four signals are collected using a polarizer combiner with a 0° polarization angle. The combined signals pass through optical fiber with a 70 km length. In the conventional system Figure 1(b), the state of polarization of each transmitted channel is 0°. Two types of optical fiber were used such as SMF and DSF and the standard parameters of each one were in Table 1. At the receiver, the signal is demultiplexed. The signal is detected by a PIN photodiode for direct detection. It is then passed through the low-pass Bessel filter. Finally, the signal is then connected directly to the system performance analyzer, which is used to generate the graph.

The nonlinear light amplitude ENL describes the FWM light; FFWM = *F*_*i*_ + *F*_*j*_ − *F*_*k*_. The total nonlinear amplitude is [10]:

(1) ENL=η|E1(0)||E2(0)||E3(0)|·(〈Sk ∣ Sj〉|Si〉+〈Sk ∣ Si〉|Sj〉),

where |*E*_*j*_(0)|  (*j* = *A*, *B*, *C*) are the amplitudes at *z* = 0.

Relative polarization states can be represented by normalized Jones vectors |*S*〉*j*, which are assumed to be maintained throughout the fiber. The orthogonal polarization effect on FWM efficiency can be classified into the following cases.

(1) For all waves identically polarized, 〈*S*_*i*_ | *S*_*j*_〉 *i* ≠ *j* = 1, the value of *X*_111*r*_^2^ = 1, and (1), for *i* and *j*, can rewritten as
  (2) $$\begin{array}{c} E^{\text{NL}}=2\eta \left|E_{1}\left(0\right)\right|\left|E_{2}\left(0\right)\right|\left|E_{3}\left(0\right)\right|,\\ {\left|E^{\text{NL}}\right|}^{2}=4\eta {\left|E_{1}(0)\right|}^{2}{\left|E_{2}(0)\right|}^{2}{\left|E_{3}\left(0\right)\right|}^{2}. \end{array}$$
(2) For the case of two waves being copolarized and the third being orthogonally polarized (|*S*_*i*_〉 = |*S*_*j*_〉⊥|*S*_*k*_〉); this means 〈*S*_*k*_ | *S*_*j*_〉 = 0,  〈*S*_*k*_ | *S*_*i*_〉 = 0, with the value of *X*_111*r*_^2^ = 0.
(3) In the case of |*S*_*i*_〉 = |*S*_*k*_〉⊥|*S*_*j*_〉, both 〈*S*_*i*_ | *S*_*j*_〉 = 0,  〈*S*_*i*_ | *S*_*k*_〉 = 1; in this case the value of *X*_111*r*_^2^ = 1/4, so the square of the nonlinear amplitude now becomes
  (3) $$\begin{array}{c} {\left|E^{\text{NL}}\right|}^{2}=\eta {\left|E_{1}\left(0\right)\right|}^{2}{\left|E_{2}\left(0\right)\right|}^{2}{\left|E_{3}\left(0\right)\right|}^{2}. \end{array}$$

In a WDM system, the power transferred to new frequencies due to FWM after light propagation within a distance *L* in the fiber can be estimated using equation [11]:

(4) $$\begin{array}{c} P_{\text{FWM}}=\eta_{n}\times \frac{1024\pi^{6}}{n_{r}^{4}\lambda^{2}C^{2}}{\left(\frac{{DX}_{111}L_{\text{eff}}}{A_{\text{eff}}}\right)}^{2}\left(P_{i}P_{j}P_{k}\right)e^{-\alpha L}, \end{array}$$

where *P*_*i*_, *P*_*j*_, and *P*_*k*_ are the input power values at central frequencies *f*_*i*_, *f*_*j*_, and *f*_*k*_, respectively. *D* is the degeneracy factor that is equal to 3 for two-tone and 6 for three-tone systems, *X*_111_ is third-order susceptibility that is equal to 6 × 10^−15^ (m^3^/w.s), *A*_eff_ is the effective area, *C* is the speed of light, *λ* is the laser wavelength, *α* is the fiber loss coefficient, *L* is the total fiber length, *n*_*r*_ is the refractive index of the fiber, and *L*_eff_ is the nonlinear effective length that can be calculated using the following equation:

(5) $$\begin{array}{c} L_{\text{eff}}=\frac{\left(1-e^{-\alpha L}\right)}{\alpha }. \end{array}$$

The efficiency (*η*) of four-wave mixing is given by [3]

(6) $$\begin{array}{c} \eta =\frac{\alpha^{2}}{\alpha^{2}+\Delta \beta^{2}}\left(1+\frac{4e^{-\alpha L}\sin^{2}\left(\Delta \beta L/2\right)}{{\left[1-e^{-\alpha L}\right]}^{2}}\right), \end{array}$$

where Δ*β* represents the phase mismatch and may be expressed in terms of signal frequency differences:

(7) Δβ=2πλ2c|fi−fk||fj−fk| ×(DC+dDdλ(λ22c)(|fi−fk|+|fj−fk|)),

where *D*_*C*_ is the fiber chromatic dispersion and *dD*/*dλ* is a derivative dispersion coefficient of the optical fiber. The right term of (6) and (7) has small and negligible values. The general equation of FWM power can be summarized as follows:

(8) PFWM=1024π6n4λ2C2(DgX111LeffAeff)2(PiPjPk) ×e−αLα2cα2+2πDc(Δfik)(Δfjk),

where (Δ*f*_*ik*_, Δ*f*_*jk*_) is the channel spacing.

Under the effect of polarization, FWM efficiency becomes

(9) $$\begin{array}{c} \eta_{\text{FWM}(\text{polirization})}=\frac{1}{N}\times \eta_{n}\times X_{111r}^{2}, \end{array}$$

where *η*_FWM(polarized)_ is FWM efficiency attained by polarization technique. *X*_1111*r*_ is a factor that represents polarization dependency of the FWM process and changes from 0 to 1 according to SOP between channels, as shown in (1) to (3).

*N* is the total number of channel and *η*_*n*_ is the FWM efficiency in the conventional system.

Using (8), FWM efficiency (*η*_*n*_) can be rewritten as follows:

(10) $$\begin{array}{c} \eta_{n}=\frac{\alpha^{2}}{c\alpha^{2}+2\pi D_{C}\left(\Delta f_{ik}\right)\left(\Delta f_{jk}\right)}. \end{array}$$

By substituting (10) into (9), we can derive (11) as the following:

(11) $$\begin{array}{c} \eta_{\text{FWM}(\text{polirized})}=\frac{1}{N}\times \frac{X_{111r}^{2}\times \alpha^{2}}{c\alpha^{2}+2\pi D_{C}\left(\Delta f_{ik}\right)\left(\Delta f_{jk}\right)}. \end{array}$$

With the polarization effect, FWM power in (8) can be modified as follows:

(12) PFWM(poarized)=1024π6n4λ2C2(DX111LeffAeff)2(PiPjPk)e−αL ×ηFWM(polarized).

By substituting (11) into (12), the general FWM power is as follows:

(13) PFWM(polirized)=1024π6n4λ2C2(DX111LeffAeff)2(PiPjPk)e−αL ×X111r2×α2N×(cα2+2πDC(Δfik)(Δfjk)).

In the Gaussian approximation, [8, 9], error probability is written as

(14) $$\begin{array}{c} P_{e}=\frac{1}{\sqrt{2\pi }}\overset{\infty }{\underset{Q}{\int }}\exp\left[-\frac{t^{2}}{2}\right]dt. \end{array}$$

To calculate system performance under the effect of FWM, shot, and thermal noises, we used the following equations:

(15) $$\begin{array}{c} Q=\frac{KP_{S}}{\sqrt{N_{\text{th}}+N_{\text{sh}}+2K^{2}Ps^{2}}C_{\text{IM}}^{\left(m\right)}+\sqrt{N_{\text{th}}}}, \end{array}$$

(16) $$\begin{array}{c} C_{\text{IM}}^{\left(m\right)}=\frac{1}{8}\sum_{\text{I}}\frac{P_{ijk}}{P_{s}}+\frac{1}{4}\sum_{\text{III}}\frac{P_{iik}}{P_{s}}, \end{array}$$

where *Q* is the *Q* factor, *C*_IM_^(*m*)^ is the effective FWM crosstalk in intensity modulation-direct modulation transmission, *P*_*ijk*_ is the FWM power generated at frequency fs from a frequency combination satisfying *f*_*i*_ + *f*_*j*_ − *f*_*k*_ = *f*_*s*_, where *f*_*k*_ = *f*_*s*_, *P*_*iik*_ is the FWM power at identical *i* and *j*, where *i* = *j* ≠ *k*, *P*_*s*_ is the received power at the receiver, *e* is the electron charge (1.6 × 10^−19^ *C*), *N*_th_ is thermal noise, and *N*_sh_ is the shot noise.

To calculate the received power and to achieve a given BER = 10^−9^, *Q* = 6, without FWM, and *C*_IM_^(*m*)^ = 0, (15) is modified as follows:

(17) $$\begin{array}{c} 2K^{2}P_{s}^{2}C_{\text{IM}}^{\left(m\right)}+N_{\text{th}}+N_{\text{sh}}=\frac{K^{2}P_{s}^{2}}{Q^{2}}-2\sqrt{N_{\text{th}}}\frac{KP_{s}}{Q}+N_{\text{th}},\\ P_{S0}=\frac{Q^{2}}{K}\left[2Be+2\frac{\sqrt{N_{\text{th}}}}{Q}\right]. \end{array}$$

The effect of shot and thermal noises can be neglected because these noises have smaller values than those of FWM noise. *Q* can be obtained using (15) as follows:

(18) $$\begin{array}{c} Q=\frac{KP_{S}}{\sqrt{2K^{2}Ps^{2}}C_{\text{IM}}^{\left(m\right)}},\\ Q^{2}=\frac{K^{2}P_{s}^{2}}{2K^{2}P_{S}^{2}C_{\left(\text{IM}\right)}^{\left(m\right)}}=\frac{1}{2C_{\left(\text{IM}\right)}^{\left(m\right)}},\\ Q=\sqrt{\frac{1}{2C_{\left(\text{IM}\right)}^{\left(m\right)}}}. \end{array}$$

BER is calculated using the following equation:

(19) $$\begin{array}{c} \text{BER}=0.5\times \mathrm{erfc}\left[\frac{Q}{\sqrt{2}}\right]. \end{array}$$

## 3. Analysis Results and Discussions

The proposed polarization technique was compared with the conventional method (without using the polarization) and examined with SMF and DSF. The comparison was conducted at an input power range of 2 dBm to 14 dBm as follows.

### 3.1. Effect of Proposed Technique on FWM Behavior and BER Using SMF

**Figure 2:**
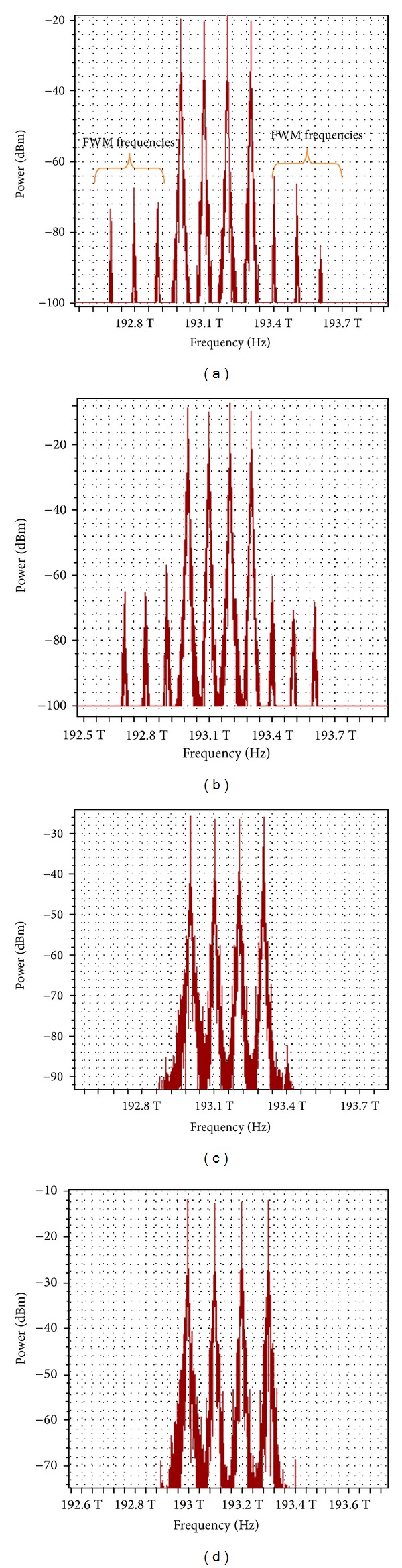
Optical spectrum comparison after 70 km SMF (a) without polarization technique at input power of 2 dBm, (b) without polarization technique at input power of 14 dBm, (c) with polarization technique at input power of 2 dBm, and (d) with polarization technique at input power 14 dBm.

The simulation for a standard single-mode optical fiber ITU-T G.652 was conducted according to the industrial environment protocol in Table 1. Figures 2(a)–2(d) illustrate the optical spectrum over a 70 km optical fiber. Decreasing the input power can decrease the FWM effects. In the absence of the polarization technique, the FWM power was −56 dBm at an input power of 14 dBm, while it was −64 dBm at a 2 dBm input power. With our proposed technique, the FWM numbers and power are dramatically reduced. The FWM power decreased to less than −82 dBm at a 2 dBm input power and to −68 dBm at a 14 dBm input power.

**Figure 3:**
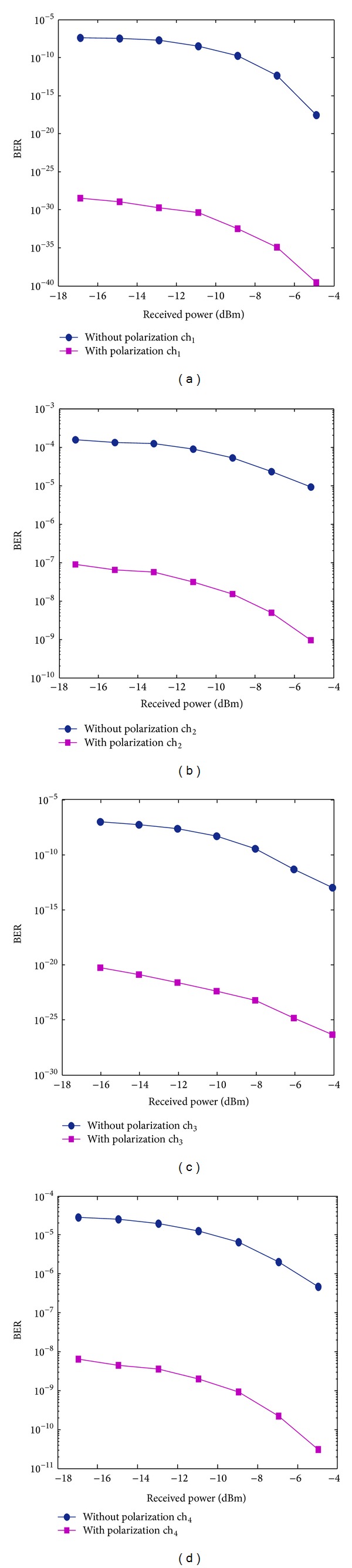
Received power versus BER in the presence and absence of the polarization techniques at (a) ch_1_, (b) ch_2_, (c) ch_3_, and (d) ch_4_.

**Figure 4:**
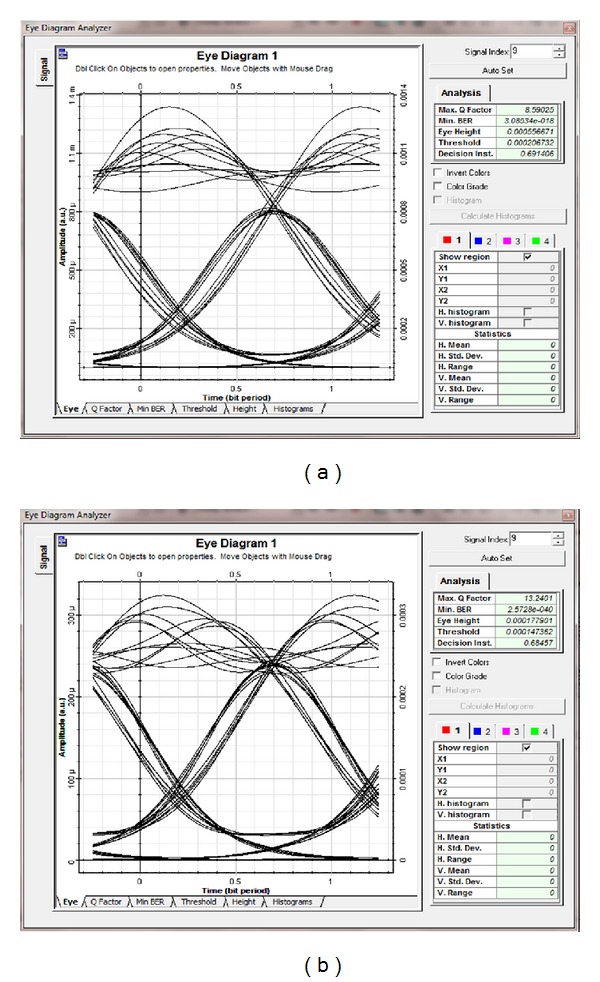
Optimum eye diagram performance of SMF using ch_1_ (a) without polarization technique and (b) with polarization. Both at *P*_in_ = 14 dBm.

Figures 3(a)–3(d) reveal that the BER in all channels improved with the polarization technique compared with the nonuse of the polarization technique. The BER values at ch_1_, ch_2_, ch_3_, and ch_4_ without the polarization technique were 3.09 × 10^−18^, 9.35 × 10^−6^, 1.11 × 10^−13^, and 4.69 × 10^−7^ at a received power of −4.9 dBm, respectively. When the polarization technique was used, the system performance, respectively, yielded the minimum BER values of 2.57 × 10^−40^, 9.35 × 10^−10^, 4.39 × 10^−27^, and 3 × 10^−11^. Figures 4(a)-4(b) compare the optimum eye diagram with the polarization technique and that without the polarization technique. The former was higher and more optimized than the latter. For ch_1_, the former was more open (BER of 2.57 × 10^−40^) than the latter (BER of 3.09 × 10^−18^) at *P*_in_ = 14 dBm.

### 3.2. Effect of Proposed Technique on FWM Behavior and BER Using DSF

**Figure 5:**
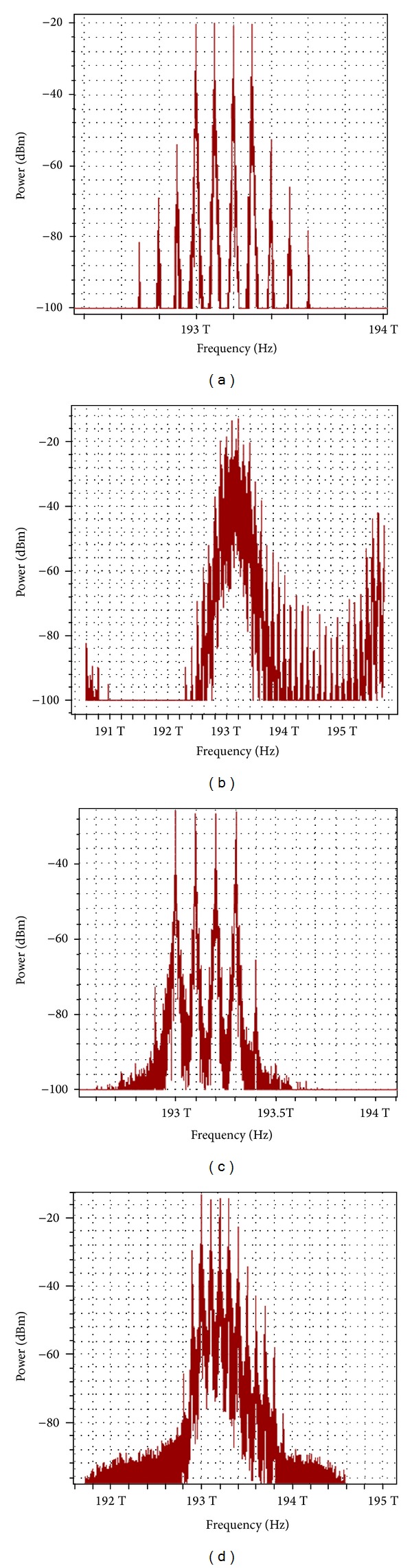
Optical spectrum comparison after 70 km DSF (a) without polarization technique at input power of 2 dBm, (b) without polarization technique at input power of 14 dBm, (c) with polarization technique at input power of 2 dBm, and (d) with polarization technique at input power 14 dBm.

For further investigation, the proposed polarization technique was tested with DSF, using the standard parameters in Table 1. In the DSF, FWM can strongly influence the transmission performance, because most of the FWM interaction occurs near zero dispersion wavelengths. At a high input power, the FWM crosstalk increased dramatically and superimposed with the transmitted channels.  Figure 5 shows that for the conventional system at a 2 dBm input power, the FWM power was −52 dBm. At a high input power of 14 dBm, the FWM power significantly increased with the number of FWM interfacing with channels, and the FWM power was about −20 dBm. With the proposed polarization technique, most of the FWM frequencies were canceled because the interaction between multiple optical channels that pass through the same fiber reduced, which suppressed the FWM. At 2 and 14 dBm input powers, the FWM powers were −69 and −25 dBm, respectively.

**Figure 6:**
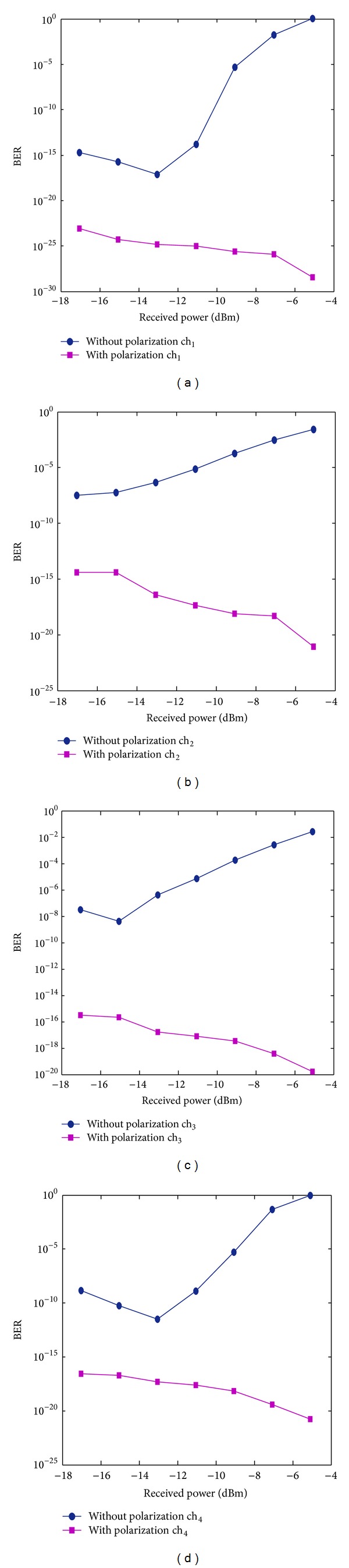
Received power versus BER in the presence and absence of the polarization techniques using DSF at (a) ch_1_, (b) ch_2_, (c) ch_3_, and (d) ch_4_.

The overlapping between the transmitted channel and the FWM crosstalk was translated into a distorted signal and detected by the receiver, which led to the significant degradation of the system performance.  Figure 6 show the BER pattern using DSF, with and without the polarization technique. With lower input power, BERs decreased with increasing received signal power. At a received power of −17.03 dBm, the BERs at ch_1_, ch_2_, ch_3_, and ch_4_ are 3.4 × 10^−15^, 4 × 10^−8^, 3 × 10^−8^, and 1.6 × 10^−9^, respectively. With the polarization technique, the BERs are respectively 7.3 × 10^−24^, 3.9 × 10^−15^, 3 × 10^−16^, and 2.8 × 10^−17^.

With the increase of the input power, the FWM power also increased and more FWM crosstalk superimposed with channels, which affected the transmitted channel. BER increased as the received signal power decreased and the noise power increased. For example, at a high input power of 14 dBm (−13.84 dBm received power), the BERs were 1, 0.05, 0.033, and 1 at ch_1_, ch_2_, ch_3_, and ch_4_ with the conventional technique, respectively. The system performance drastically improved in the presence of the polarization technique, where the BER values were respectively 3.4 × 10^−29^, 8 × 10^−22^, 1.6 × 10^−20^, and 1.8 × 10^−21^. Based on these findings, the polarization approach introduces a dramatic durability for reducing the FWM crosstalk, even at high input power and low dispersion. In terms of the eye diagram, the first channel revealed a high and optimum eye diagram in the proposed technique than in the conventional one.

**Figure 7:**
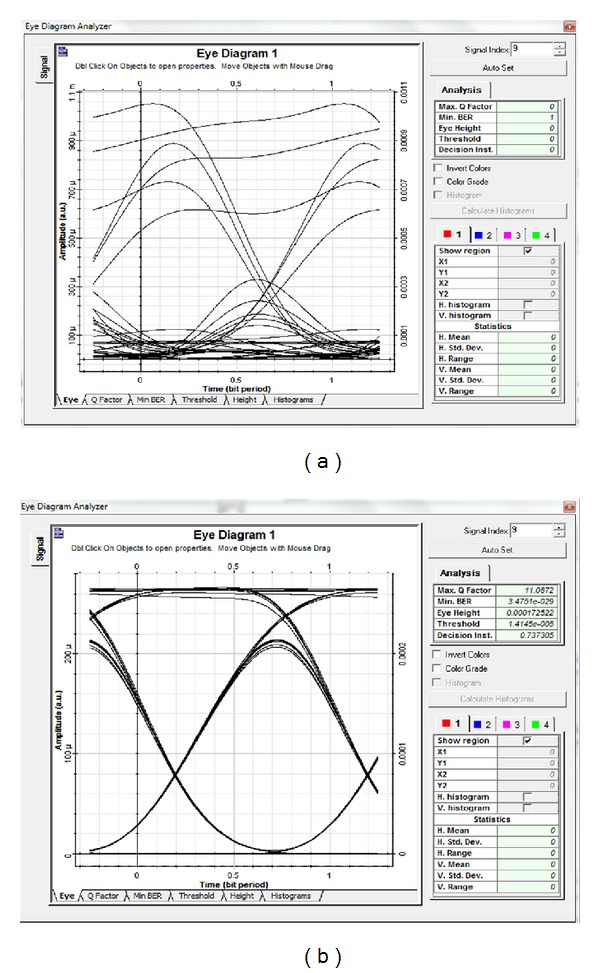
Optimum eye diagram performance of DSF using ch_1_ (a) without polarization technique and (b) with polarization. Both at *P*_in_ = 14 dBm.

Figures 7(a)-7(b) illustrate the eye diagram using DSF for both techniques at a 14 dBm input power. The polarization technique was superior to the conventional technique at high input power values.

## 4. Conclusion

In this paper, we propose an efficient approach for reducing the transmission limitation caused by the FWM in a WDM system by using the pairing combinations of differently linear-polarized optical signals. The FWM behavior and system performance were evaluated with the proposed technique under the input power parameter and using two kinds of optical fiber, namely, SMF and DSF. The FWM powers were suppressed to less than −68 and −25 dBm at a 14 dBm input power in the presence of this approach using the SMF and DSF, respectively. The polarization technique also enhanced the BERs in the range of 2.57 × 10^−40^ and 3.47 × 10^−29^ at received powers of −4.90 and −13.84 dBm for SMF and DSF, respectively. The findings prove that the polarization approach significantly reduced the FWM crosstalk through fiber transmission. The obtained results also show that in the existence of the polarization approach, the FWM crosstalk has no dangerous effect, even with a high input optical power of 14 dBm.

## Figures and Tables

**Table 1:** System simulation parameters.

Parameter	Unit	Values
Fiber length, *L*	km	70 for SMF
Input power, *P*_*i*_	dBm	Varied from 2 to 14 dBm with step 2 dBm
Input frequencies, *f*_*i*_, *f*_*j*_ and *f*_*k*_	THz	193 to 193.3
Channel spacing, Δ*f*	GHz	100
Standard SMF G.652		
Dispersion, *D*_*c*_	ps/nm·km	17
Cross effective area, *A*_eff_	*μ*m^2^	80
Dispersion slope	ps/nm^2^·km	0.07
Standard DSF G.653		
Dispersion, *D*_*c*_	ps/nm·km	0.3
Cross effective area, *A*_eff_	*μ*m^2^	50
Dispersion slope	ps/nm^2^·km	0.075
Degeneracy factor, *D*_*g*_	—	6
Third order susceptibility, *X*_111_	m^3^/w·s	6 × 10^−15^
Refractive index, *n*	—	1.48
Speed of light, *c*	(m/s)	3 × 10^8^
Attenuation factor	(dB/km)	0.2
Number of channel	—	4
*K*, detector responsively	A*∖*W	0.8
Total data rate	Gb/s	40
Optical bandwidth, *B*	MHz	622
